# Linguistic and psychometric validation of the Chinese version of the Glaucoma Quality of Life-15 (GQL-15-CHI): a cross-sectional study

**DOI:** 10.1186/1477-7525-11-188

**Published:** 2013-11-05

**Authors:** Chuandi Zhou, Jing Yao, Shaohong Qian, Peixia Wu

**Affiliations:** 1 Department of Ophthalmology, Eye and ENT Hospital, Shanghai Medical College, Fudan University, Shanghai, China; 2 Department of Nursing, Eye and ENT Hospital, Shanghai Medical College, Fudan University, Shanghai, China

## Abstract

**Background:**

Maintaining glaucoma patients’ quality of life (QoL) has become one of the most important goals for treatments. The purpose of this study is to develop a Chinese version of Glaucoma Quality of Life-15 item Questionnaire (GQL-15-CHI), and examine its psychometric properties.

**Methods:**

The Glaucoma Quality of Life-15 item Questionnaire (GQL-15) was translated and culturally adapted into Chinese, and administered to glaucoma patients recruited from Shanghai Eye and ENT Hospital. Visual functions: habitual-corrected visual acuity (HCVA), intraocular pressure (IOP), and mean defect (MD) of visual field) were assessed through clinical examination by professionals. Sociodemographic and other clinical data were collected via interviews and chart review. According to Nelson’s glaucoma staging system, patients were stratified as mild, moderate, and severe visual field loss (VFL). The psychometric properties, including internal consistency, test-retest reliability, item-scale correlations and factor analysis were conducted. The divergent validity was assessed through bilateral comparisons of the GQL-15-CHI composite and subscale scores between patients of different VFLs after controlling for potential confounders.

**Results:**

A total of 508 glaucoma patients were recruited (male: 265, female: 243). The mean age was 55.41 years. The Cronbach’s α coefficients ranged from 0.75 to 0.91 for the subscales. The test-retest reliability, as estimated by the intraclass correlation coefficients, were above 0.70 for all subscales. Statistically significant differences were showed in the GQL-15-CHI summary and subscale scores after controlling for sociodemographic and clinical confounders (P < 0.05) among patients with different VFLs.

**Conclusion:**

The GQL-15-CHI showed psychometric properties comparable to those of the original English version, and thus could be used as a reliable and valid tool for assessment of QoL in Chinese glaucoma patients.

## Background

Glaucoma is the second leading cause of blindness worldwide. It was estimated that about 8.4 million people were blind due to glaucoma worldwide in the year of 2010, and more than one-fourth of them lived in China [1,2]. Apart from traditional measures, such as intraocular pressure (IOP), perimetry, visual acuity, in recent years, assessment of quality of life (QoL) is being increasingly recognized as a critical measurement in monitoring and evaluating the effectiveness of different treatments of glaucoma [3-7]. “QoL” is a subjective perception of well-being and wholeness. It is a broad concept incorporating the patient’s perspective of his or her health. At the same time, it reflects the difference between the patients’ expectations and their present status [8].

Glaucoma may impact patient’s QoL in several aspects: the visual effects of glaucoma (decreased visual field and ultimately visual acuity), the psychological burden caused by diagnosis (fear of blindness [9], fear of affliction to other members of the family, anxiety and depression [10]), the potential side effects of treatment (medical and/or surgical), and the financial burden (cost of visits and therapies [11], loss of income because of absenteeism from work). Even patients in the early stages of glaucoma experience deficits in QoL associated with self-perceived visual dysfunction [3,4,12,13]. Therefore, maintaining a patient’s QoL has always been an important goal for glaucoma treatments.

Compared to the generic patient-reported outcome measures (PROMs), such as the National Eye Institute Visual Function Questionnaire 25 items [14,15], glaucoma-specific questionnaires attached more importance to patients’ visual field loss (VFL) [4,16,17]. The Glaucoma Quality of Life-15 (GQL-15) is one of them, and has been proved to perform well among glaucoma patients [4].

The GQL-15 was extracted from the original 62 items according to their relationship to visual field loss [13]. As this instrument is short and easy to use, it is accepted worldwide. Various studies have consistently demonstrated that GQL-15 score has a strong correlation with objective visual measures [18-21]. However, the results are also affected by different demographic or other factors, and the GQL-15 scores vary from region to region [4,18,19,21]. At present, there are no validated Chinese versions of QoL questionnaires specific to glaucoma patients, thereby limiting the international comparison of outcomes of treatment for Chinese glaucoma patients [22]. Based on the interest in this field, we set out to translate and validate the GQL-15 to use it in Chinese glaucoma patients. Because transcultural adaptation of a questionnaire requires that the questionnaire’s psychometric properties are reestablished within the new cultural and linguistic context [23,24], the aim of this study was to develop the Chinese version of GQL-15 (GQL-15-CHI) and to evaluate its psychometric properties in Chinese glaucoma patients.

## Methods

### Patients

This study was approved by the Institutional Review Board of Fudan University and adhered to the tenets of the Declaration of Helsinki. Patients with glaucoma were recruited from both office practice and wards in Shanghai Eye and ENT Hospital from January to August 2012. Informed consents were obtained from all patients.

Eligibility criteria were Chinese-speaking adult patients (18 years or older) with a glaucoma diagnosis more than 6 months prior to enrollment [9,25]. Glaucoma was diagnosed based on glaucomatous disc cupping and reproducible visual field damage in one or both eyes. Patients with primary open angle glaucoma (POAG), normal tension glaucoma (NTG), primary angle-closure glaucoma (PACG), and secondary glaucoma (SG) were included in this study.

The exclusion criteria were: (1) major psychiatric problems; (2) the patient could not speak, read or understand Mandarin-Chinese; (3) current use of any medication with possible side effects of a psychiatric disorder or cognitive impairment that might affect the psychological assessment, e.g., systemic use of beta blockers [26,27]; (4) incisional eye surgery within the previous three months or laser treatment within the previous one month; (5) disability in visual field testing due to causes other than glaucoma (e.g., cognitive impairment such as dementia caused by Alzheimer’s disease); (6) other severe vision-impaired eye diseases (e.g., cataracts (Lens Opacities Classification System III [28] grade 2 or more) and wet age-related macular degeneration).

A trained interviewer explained the purpose of this study to glaucoma patients, and they participated voluntarily without any additional compensation. Of the 552 patients who met the inclusion criteria, 36 declined to participate in our study due to time constraints. Of the 516 subjects who agreed to participate, 8 patients were excluded from the analysis because of incompletely answered questionnaires, leaving a final sample size of 508.

All eligible patients were divided into three groups by the severity of central visual field impairment according to Nelson’s glaucoma staging system [4]: mild (unilateral loss with less than half of the visual field lost), moderate (unilateral loss with more than half of the visual field lost, or bilateral loss with less than half of the visual field lost in each eye), and severe (bilateral loss, more than half of the visual field lost in both eyes).

### Data collection

All interviews were performed by a trained interviewer who was not involved in the ophthalmic examination. Sociodemographic data including age, gender, living situation, family history of glaucoma, duration of glaucoma, education level, and personal monthly income, were obtained through face-to-face interviews. Questionnaires were then completed before the examination to ensure that the clinical data would not influence the subjective responses. Twenty patients were retested after two weeks to determine the test-retest reliability of the questionnaire.

All eligible patients received comprehensive ophthalmic examinations, including habitual-corrected visual acuity (HCVA) using the Snellen visual acuity chart, intraocular pressure (IOP) with a Goldmann applanation tonometer, a complete ocular examination with slit-lamp biomicroscopy, direct and indirect fudus ophthalmoscopy, and a central 30° visual field evaluation using automated static perimetry (OCTOPUS 900, Haag-Streit Eye Suite, Switzerland). Only “reliable” visual fields were used, as defined by a reliability factor (RF) not exceeding 15%. Clinical indices, such as mean defect (MD) of visual field, number of IOP-lowering medications used, anti-glaucoma laser and surgeries in treatment history, period on topical beta blocker eye drops were also collected by chart review. The data were labeled with serial numbers and analyzed in a way to protect patient privacy.

### GQL-15 questionnaire

The GQL-15 [4] consists of 15 vision-related items, which primarily cover four aspects: central and near vision (two items), peripheral vision (six items), glare and dark adaptation (six items), and outdoor mobility (one item). Summary scores represented the sum of item-level response scores, with higher scores indicating poorer QoL. The item-level responses for each factor were coded on a scale from 1 (no difficulty) to 5 (severe difficulty), and 0 represented “abstinence from activity due to nonvisual reasons”. For subscale scores, the item-level responses were scored on a numerical interval scale ranging from 0 (no difficulty) to 100 (severe difficulty). Subscale scores were calculated for each factor by averaging the sum of scores generated for the item-level responses. Higher subscale scores represented greater difficulty with vision-related activities and poorer QoL.

### Linguistic validation of GQL-15-CHI

The GQL-15-CHI was developed according to standard methods that have been adopted internationally [29], including forward translation, back translation, examination of the translation quality by bilingual speakers, and a pilot test among 20 patients. The wording of two items was changed to better adapt them to the Chinese lifestyle and culture. After reviewing the content of the translated questionnaire by one glaucoma expert and one psychologist, a psychometric test was conducted.

The linguistic validation of GQL-15-CHI included six steps:

1. The GQL-15-CHI was translated from English into Chinese by two professional translators.

2. A panel (two translators and two glaucoma experts) deliberated the Chinese translations to produce a second draft of the GQL-15-CHI.

3. A third translator, who was not involved in the forward translation and was blinded to the original questionnaire, back translated the drafted GQL-15-CHI into English.

4. The back-translated GQL-15-CHI was compared with the original English version to identify any discrepancies, which were then deliberated by the panel.

5. Proper adaptation of some items was considered necessary during the translation and validation of the questionnaire in other populations [23,24,30,31]. Cognitive debriefing of the drafted GQL-15-CHI was performed on 20 patients with glaucoma to test their understanding and interpretation of the questionnaire, and two items were modified to make them better understood. Patients were frequently confused by some items, e.g. “judging distance of foot to step” in the original version, and it was revised as “can you figure out the distance between you and me or how far is that object (e.g. the door) apart from you?” Compared to the original form, this way was more natural and vivid to Chinese patients, and all participants could catch the exact meaning immediately. Moreover, another puzzled item, “seeing objects coming from the side”, and it was modified to “unable to see the objects directly at the front but being able to see objects coming from the side” in the Chinese version to increase response accuracy.

6. The final version of the GQL-15-CHI was established after minor revisions based on the outcome of the cognitive debriefing.

### Statistical analyses

HCVA was converted to logarithm of the minimum angle of resolution (logMAR). All statistical analyses were performed using SAS software version 9.2 (SAS Institute, Inc., Cary, NC).

### Descriptive analyses

Descriptive statistics were used to determine the distribution of sociodemographic and clinical characteristics, and were reported as mean ± standard deviation (SD) or proportion (%).

### Reliability

Cronbach’s α coefficient was used as an index of internal consistency for each subscale. The optimal range of Cronbach’s α was above 0.70. To quantify test-retest reliability, intraclass correlation coefficients were used [32]. Reliability coefficients above 0.70 were considered satisfactory [33]. To further determine scale homogeneity, the item-cale correlation coefficient was calculated and a coefficient greater than 0.40 was considered acceptable [34]. The percentage of item response at the ceiling (lowest subscore) and floor (highest subscore) of the GQL-15-CHI were also calculated.

### Validity

Five experts (three glaucoma specialists and two statisticians) were invited to evaluate the face validity of the final version of GQL-15-CHI. Exploratory factor analysis after varimax rotation was used to further evaluate validity. To test the discriminatory power of the questionnaire, first, univariate comparisons of sociodemographic and clinical data between patients of mild, moderate and severe VFL were made using either analysis of variance (continuous factors) or Chi-square test (categorical factors). Moreover, post hoc pairwise comparisons between different categories of glaucoma severities were carried out using Turkey’s honestly significant difference test. The significant variates were considered as confounders in linear regression analyses. After orderly controlling for these potential confounders, bilateral comparisons of the GQL-15-CHI composite and subscale scores were made between patients of mild, moderate and severe VFLs.

## Results

### Demographics

A total of 508 glaucoma patients were included in this study. The mean age was 55.41 ± 15.23 years with a range of 18–88 years, and 265 (52.17%) patients were male. The mean duration of glaucoma was 5.06 ± 6.31 years, ranging from 0.5 to 49.0 years. Age, education level, duration of glaucoma, HCVA (both in the better eye and the worse eye), MD (both in the better eye and the worse eye), and higher IOP of both eyes were all found to be significantly different among patients with different VFLs. No significant differences were found with respect to gender, living situation, or family history of glaucoma among the three groups. The overall sociodemographic and clinical characteristics of the patients are summarized in Table 1.

**Table 1:** Sociodemographic and clinical characteristics of the Chinese patients with glaucoma

**Variable**	**Total**	**Mild VFL**	**Moderate VFL**	**Severe VFL**	***P***	***P***_**1**_	***P***_**2**_	***P***_**3**_
Number of participants	508	133(26.18)	306(60.24)	69(13.58)				
Age (years)	55.41 ± 15.23	50.30 ± 16.07	57.22 ± 14.35	57.22 ± 15.32	<.01	<.01	0.01	1.00
Male gender	265(52.17)	65(48.87)	163(53.27)	37(53.62)	0.68	0.40	0.52	0.96
Duration of glaucoma (years)	5.06 ± 6.31	3.53 ± 4.32	5.14 ± 5.62	7.63 ± 10.40	<.01	0.04	<.01	0.01
Education					0.01	0.02	<.01	0.01
Below primary/primary (≤6 years)	68(13.39)	11(8.27)	39(12.74)	18(26.09)				
Secondary (7–13 years)	253(49.80)	60(45.11)	160(52.29)	33(47.83)				
Tertiary (>13 years)	187(36.81)	62(46.62)	107(34.97)	18(26.09)				
Living situation					0.76	0.57	0.48	0.74
Family	471(92.72)	125(93.98)	283(92.48)	63(91.30)				
Alone	37(7.28)	8(6.02)	23(7.52)	6(8.70)				
Economic status (RMB/month)					<.01	0.34	<.01	<.01
<3000	65(12.80)	11(8.27)	36(11.77)	18(26.09)				
3000-5000	215(42.32)	57(42.86)	130(42.48)	28(40.58)				
>5000	228(44.88)	65(48.87)	140(45.75)	23(33.33)				
Economic burden					0.01	0.27	<.01	0.04
Light	124(24.41)	35(26.31)	74(24.18)	15(21.74)				
Moderate	255(50.20)	73(54.89)	157(51.31)	25(36.23)				
Heavy	129(25.39)	25(18.80)	75(24.51)	29(42.03)				
Positive glaucoma family history	107(21.06)	30(22.56)	59(19.28)	18(26.09)	0.40	0.43	0.58	0.21
Type of glaucoma					0.05	0.03	0.28	0.23
PACG	178(35.04)	35(26.32)	121(39.54)	22(31.88)				
POAG and NTG	264(51.97)	76(57.14)	147(48.04)	41(59.42)				
Secondary glaucoma	66(12.99)	22(16.54)	38(12.42)	6(8.70)				
LogMAR HCVA in better eye	0.20 ± 0.37	0.06 ± 0.13	0.17 ± 0.25	0.59 ± 0.73	<.01	<.01	<.01	<.01
LogMAR HCVA in worse eye	0.80 ± 0.99	0.24 ± 0.29	0.86 ± 1.00	1.60 ± 1.15	<.01	<.01	<.01	<.01
MD in better eye	7.02 ± 6.62	1.20 ± 1.11	6.49 ± 3.53	20.57 ± 3.86	<.01	<.01	<.01	<.01
MD in worse eye	14.53 ± 8.79	4.91 ± 3.59	16.51 ± 7.36	24.32 ± 3.45	<.01	<.01	<.01	<.01
Period on using topical beta blocker (years)	1.04 ± 2.31	0.55 ± 1.37	1.00 ± 1.91	2.17 ± 4.25	<.01	0.17	<.01	<.01
Number of antiglaucoma medication used	1.57 ± 1.10	1.33 ± 0.93	1.54 ± 1.08	2.19 ± 1.25	<.01	0.17	<.01	<.01
Number of glaucoma surgery in treatment history	0.84 ± 1.07	0.56 ± 1.03	0.90 ± 1.02	1.12 ± 1.23	<.01	<.01	<.01	0.37
Number of laser intervention in glaucoma treatment	0.36 ± 0.78	0.39 ± 0.81	0.36 ± 0.74	0.30 ± 0.90	0.76	1.00	1.00	1.00
Higher IOP of both eyes	20.32 ± 9.49	18.08 ± 5.51	20.54 ± 9.86	23.68 ± 12.41	<.01	0.04	<.01	0.04

Data are presented as mean ± SD or n(%); *P*_1_: mild vs. moderate; *P*_2_: mild vs. severe; *P*_3_: moderate vs. severe. VFL: visual field loss; PACG: primary angle closure glaucoma; POAG: primary open-angle glaucoma; NTG: normal tension glaucoma; LogMAR: logarithm of the minimum angle of resolution; HCVA: habitual corrected visual acuity; MD: mean defect of visual field; IOP: intraocular pressure.

### Reliability

The Cronbach’s α coefficient (internal consistency index) was 0.96 for the overall analyses and ranged from 0.75 to 0.91 for the subscales (Table 2). The test-retest reliability, as estimated by the intraclass correlation coefficients, were greater than 0.70 for all subscales (Table 2). These two tests were not applied to the subscale of outdoor mobility, which was composed of only one item. The item-scale correlation coefficient was calculated to determine scale homogeneity and showed that the coefficients were all above 0.40: item-total scale correlation coefficients ranged from 0.56 to 0.81 and item-subscale correlation coefficients ranged from 0.54 to 1.00 (Table 2). Floor effect was not found in any of the subscales. However, the percentage of subjects scoring at the ceiling was over 20% in three subscales (Central and near vision, Peripheral vision and Outdoor mobility).

**Table 2:** Reliability analysis of Chinese version of Glaucoma Quality of Life-15 item Questionnaire (GQL-15-CHI)

**Subscale and item**	**Item-total scale correlation**	**Item-subscale correlation**	**Cronbach α**	**Intraclass correlation coefficient**	**Ceiling** **n (%)**	**Floor** **n (%)**
Central and near vision			0.75	0.73 (0.65-0.79).	158 (31.10)	11 (2.17)
Recognizing faces	0.64	0.58				
Reading newspapers	0.69	0.58				
Peripheral vision			0.91	0.91 (0.90-0.92)	143 (28.15)	3 (0.59)
Seeing objects coming from the side	0.61	0.61				
Walking on uneven ground	0.78	0.74				
Tripping over objects	0.69	0.72				
Judging distance of foot to step	0.63	0.65				
Walking on steps/stairs	0.76	0.74				
Bumping into objects	0.69	0.73				
Glare and dark adaptation			0.90	0.90 (0.88-0.91)	54 (10.63)	1 (0.20)
Walking after dark	0.81	0.80				
Seeing at night	0.78	0.77				
Adjusting to dim light	0.69	0.70				
Adjusting to bright light (glare)	0.56	0.54				
Going from light to dark room or vice versa	0.72	0.73				
Finding dropped objects	0.77	0.70				
Outdoor mobility			NA	NA	249 (49.01)	15 (2.95)
Crossing the road	0.77	1.00				

NA: not available for only one-item subscale.

### Validity

Five experts evaluated the face validity of the questionnaire, and the results showed a face validity index of 0.98. The mean summary score for the GQL-15-CHI was 28.79 ± 12.74 and the mean subscale scores were as follows: central and near vision (26.18 ± 26.56), peripheral vision (18.03 ± 21.37), glare and dark adaptation (28.19 ± 22.86), and outdoor mobility (15.06 ± 24.57). Of these four factors, the scores for glare and dark adaptation were consistently higher than the others, while the scores for outdoor mobility were the lowest.

In order to test the divergent validity, patients were classified as mild, moderate, and severe VFL according to Nelson’s glaucoma staging system. After controlling for age and gender, it showed a significant difference between the three groups in GQL-15-CHI composite and subscores (P < 0.01, not shown in Table 3). This trend was not weakened after adjustment for sociodemographic factors, which included educational level, personal monthly income, economic burden (P < 0.01, not shown in Table 3). After further adjustment for clinical indices, which included duration of glaucoma, logMAR HCVA of the better eye and the worse eye, period on topical beta blocker, number of glaucoma surgeries in treatment history, number of antiglaucoma medications used, higher IOP of both eyes, patients with increasing severity of VFL consistently had higher mean ranks of GQL-15-CHI summary and subscale scores (Table 3, P < 0.05).

**Table 3:** Divergent validity of Chinese version Glaucoma Quality of Life-15 (GQL-15-CHI) of patients with different visual field loss*

**Variables**	**Mild VFL**	**Moderate VFL**		**Severe VFL**	
		***β*** **(SE)**	***P***	***β*** **(SE)**	***P***
CHI-GQL-15 summary score	20.76 ± 6.12	28.73 ± 10.81		44.55 ± 15.33	
	Reference	4.63(1.04)	<0.01	15.43(1.62)	<0.01
		Reference		10.80(1.36)	<0.01
Central and near vision	11.18 ± 14.78	26.84 ± 24.82		52.17 ± 30.77	
	Reference	0.72(0.18)	<0.01	1.93(0.29)	<0.01
		Reference		1.21(0.24)	<0.01
Periphery vision	5.55 ± 9.13	17.42 ± 17.77		44.87 ± 28.12	
	Reference	1.65(0.44)	<0.01	6.48(0.68)	<0.01
		Reference		4.83(0.57)	<0.01
Glare and dark adaptation	14.16 ± 13.73	28.59 ± 20.62		53.44 ± 24.44	
	Reference	2.09(0.48)	<0.01	5.93(0.74)	<0.01
		Reference		3.84(0.62)	<0.01
Outdoor mobility	3.38 ± 9.62	13.48 ± 20.75		44.57 ± 35.06	
	Reference	0.17(0.08)	<0.01	1.09(0.13)	<0.01
		Reference		0.92(0.11)	0.04

The CHI-GQL-15 summary and subscale scores were presented as mean ± standard deviation; SE: standard error; VFL: visual field loss; *adjusted for age, gender, education, personal monthly income, economic burden, duration of glaucoma, logarithm of the minimum angle of resolution (logMAR) habitual-corrected visual acuity (HCVA) of the better eye and the worse eye, period on topical beta blocker, number of glaucoma surgeries in treatment history, number of antiglaucoma medication used, higher intraocular pressure of both eyes.

Exploratory factor analysis after varimax rotation was used to further evaluate validity and the results are shown in Table 4. Items were included only if they loaded on a factor and had a loading greater than 0.40. Factor analysis indicated that GQL-15-CHI was comprised of four factors explaining 68.22% of the cumulative variation.

**Table 4:** Factor loading of Chinese version Glaucoma Quality of Life-15 (GQL-15-CHI) after varimax rotation

**CHI-GQL-15 items**	**Factor 1**	**Factor 2**	**Factor 3**	**Factor 4**
11. Walking on steps/stairs	**0.91**	0.11	-0.01	0.13
10. Crossing the road	**0.90**	0.10	-0.01	0.18
4. Walking on uneven ground	**0.71**	0.06	0.33	0.09
8. Tripping over objects	0.37	**0.77**	0.16	-0.01
9. Seeing objects coming from the side	-0.01	**0.69**	-0.22	0.16
12. Bumping into objects	0.53	**0.65**	-0.08	0.03
1. Reading newspapers	0.06	**-0.74**	0.01	0.18
6. Adjusting to dim light	-0.02	-0.01	**0.78**	-0.06
7. Going from light to dark room or vice versa	-0.02	0.07	**0.75**	0.01
3. Seeing at night	0.21	-0.17	**0.69**	0.40
2. Walking after dark	0.43	-0.07	**0.69**	0.32
13. Judging distance of foot to step	0.42	0.23	**-0.46**	0.31
15. Recognizing faces	-0.02	-0.30	0.05	**0.85**
14. Finding dropped objects	0.28	0.17	0.06	**0.69**
5. Adjusting to bright light (glare)	-0.25	-0.53	-0.13	**-0.60**
Eigenvalue	4.44	2.82	1.73	1.24
Variance explained (%)	29.61	18.81	11.50	8.30

Bold numbers represents the factor loading of the items.

## Discussion

In this study, we developed a Chinese version of the GQL-15 and evaluated its psychometric properties in Chinese glaucoma patients. Overall, we demonstrated that the GQL-15-CHI was reliable, valid, and able to discriminate the severity of glaucoma.

With respect to the reliability, the overall Cronbach’s α coefficient was 0.96 in this study, which was similar to that of the original version (0.95) [4]. The Cronbach’s α coefficients for the subscales were all greater than 0.70, indicating satisfactory internal consistency of the questionnaire in the studied population. Considering that intraclass correlation coefficients greater than 0.70 are generally accepted as satisfactory, our results suggested that the GQL-15-CHI demonstrated good test-retest reliability. High item-scale and item-subscale correlations further confirmed the excellent homogeneity of the questionnaire. A ceiling effect was noted for the subscale of central and near vision, peripheral vision, and outdoor mobility. Good performance in the three subscales may indicate patients’ adaptation in handling the daily activities involving these aspects. Furthermore, GQL-15-CHI is probably to be sensitive to any worsening of QoL throughout all four domains as no floor effect was observed.

Because the GQL-15 was designed for subjective assessment of visual functions in glaucoma patients to guide the treatment, it was important for the questionnaire to have strong discriminatory power. Our results showed that the GQL-15-CHI had a good capacity to discriminate the severity of glaucoma. Greater difficulty with vision-related activities and poorer QoL were found to be highly correlated with increasing disease severity even after adjustment for all sociodemographic and clinical confounders. This finding indicated that the subjective visual function assessment could function as a complement to objective visual measures, and was informative and predictive in the follow-ups of glaucoma patients. This suggests value for the ophthalmologist in establishment of a baseline QoL upon diagnosis of glaucoma and periodically thereafter.

Nelson et al. [4] observed a marked difference in GQL-15 summary scores only between patients with mild and severe glaucoma. Onakoya et al. [19] detected statistically significant differences between patients with moderate and severe glaucoma and mild and severe glaucoma. However, in our study the differences among patients in all stages of glaucoma were statistically significant, consistent with results reported by Goldberg et al. [18]. However, after categorization, the mean summary scores for moderate and severe glaucoma in our study (28.73 and 44.55) were significantly higher than those reported by Nelson et al. [4] (22.5 and 24.9) and Onakoya et al. [19] (20.58 and 32.65). The trend among the mean summary scores for different stages of glaucoma in our study (20.76, 28.73, and 44.55) was similar to that previously found by Goldberg et al. [18] (21.7, 29.6, and 40.0). Potential explanations for this discrepancy may lie in cultural and/or social variances, varied sample sizes (China: 508; Nigeria: 132; Australia: 121; England: 47), different methods for categorization of patients, and dissimilar patient selection criteria. With respect to patient selection, different age distributions (China: 18–88 years; Nigeria: ≥40 years; Australia: ≥44 years; England: 53–81 years) and types of glaucoma (China: POAG, NTG, PACG and SG; Nigeria: POAG; Australia: POAG; England: POAG, NTG, PACG and SG) were partially responsible for the discrepancies. Moreover, Nelson et al. [4] excluded patients with progressive visual fields and visual impairment to reduce the effect of visual acuity on the study; whereas our study, Goldberg et al. [18], and Onakoya et al. [19] did not. Although Nelson’s study presented with a good homogeneity, it probably limited the diversity of disease severities.

The results of the factor analysis in our study were not fully conformed to the item distribution of the four dimensions in the original version. The explanations underlying this difference may relate to the following aspects: First and foremost, compared to the Nelson’s patient sample, we had a much larger and heterogeneous patient population. In a certain sense, the finding in our study demonstrated that the original version may not generalize to the patients of more severe glaucomatous impairment. Second, the cultural differences may still exist and result in a deviation in perceiving the items of the questionnaire, although we modified some items to make it more adaptable to Chinese patients. Third, social variance could cause a difference in the response to some specific items, e.g. for the item “crossing the road”, the traffic is crowded in China for the largest population in the world, and this is more pronounced in the metropolitans, such as Shanghai. In addition, item 13 did not fit a specific dimension, while items 2, 3 and 12 were far from being perfect. Consequently, the Chinese patients were more likely to express difficulty in response to this item. Rasch analysis, may be used as a possible method to reengineer the GQL-15-CHI to be a better-structured questionnaire in future research [35].

Although the psychometric characteristics of the GQL-15-CHI were comparable with those of the original version, the following limitations should be considered when interpreting the results of this study. First, reliance on self-reported visual symptoms may be influenced by recall bias and personality factors. Second, all patients in this study were recruited from a single tertiary institution and belonged to the Chinese Han ethnicity. Although this specialized hospital received patients from all parts of China and Han ethnicity takes up more than 91% of total Chinese population, there may still exists selection bias. In addition, we made six months as the duration cut point when recruiting participants, which can also cause a deviation in selecting the patients. Third, we did not include controls in this study, thereby limiting the conclusions that we can draw from the study regarding whether or not it can discriminate between glaucoma and other visual-impaired disease. Fourth, we did not cover all ocular examinations originally reflected by the original GQL-15 design, such as contrast sensitivity, critical flicker frequency, dark adaptation, glare disability, and stereoacuity. Lastly, this study did not carry out over time for longitudinal observation, and confirmatory factor analysis was not conducted in another sample to validate our findings. Therefore, a larger series with long-term follow-up including control groups and other parameters is needed to further validate the GQL-15-CHI in Chinese populations. Despite these limitations, this study included a large sample size. Moreover, the comprehensive ophthalmic examinations were performed by one expert and therefore avoiding the inter-observer errors.

## Conclusions

We successfully translated and adapted the GQL-15 into Chinese and demonstrated that its psychometric characteristics were comparable with the original questionnaire. In particular, the GQL-15-CHI had strong clinical discriminatory power with respect to severity of glaucoma, making it a reliable and valid tool to help patients and clinicians better manage the disease. Given its simplicity, brevity, and significant relationship with VFL, the GQL-15 will facilitate the comparison of multicenter and multilingual research.

## Abbreviations

GQL-15-CHI: Chinese version of the Glaucoma Quality of Life-15; GQL-15: Glaucoma Quality of Life-15 Item Questionnaire; QoL: Quality of life; HCVA: Habitual-corrected visual acuity; IOP: Intraocular pressure; MD: Mean defect; VFL: Visual field loss; POAG: Primary open angle glaucoma; NTG: Normal tension glaucoma; PACG: Primary angle-closure glaucoma; SG: Secondary glaucoma; RF: Reliability factor; logMAR: Logarithm of the minimum angle of resolution; SD: Standard deviation.

## Competing interests

The authors declare that they have no competing interest.

## Authors’ contributions

CZ devoted in coordination and design of this study, data analyses, drafting and revising the data. JY contributed in data analysis and drafting this article. SQ assumed the concept and design of this study and acquisition of patients. PW did the training of interviewers, and interpretation of the data. All authors read and approved the final manuscript.

## Authors’ information

Chuandi Zhou and Jing Yao are co-first author.
